# Onyx Embolization of a Meningioma with a Dysplastic Aneurysmal Anterior Cerebral Artery Vessel

**DOI:** 10.7759/cureus.776

**Published:** 2016-09-11

**Authors:** Daniel R Felbaum, Kyle Mueller, Ai-Hsi Liu, Rocco A Armonda

**Affiliations:** 1 Neurosurgery, Medstar Georgetown University Hospital; 2 Neurointerventional Radiology, Medstar Washington Hospital Center; 3 Neurosurgery, Medstar Washington Hospital Center

**Keywords:** neurointerventional procedures, tumor embolization

## Abstract

Preoperative embolization of meningiomas can be safely performed using a variety of embolic agents. Most commonly, the vascular supply from branches of the external carotid artery is the target of embolization. In our report, we detail the treatment of a patient with a parafalcine meningioma that received its supply via branches of the anterior cerebral artery. One of the feeder vessels appeared to contain a dysplastic aneurysmal dilatation of the vessel. Due to patient circumstances, embolization was performed using standard microcatheterization techniques to minimize intraoperative blood loss. We report a rare instance of endovascular treatment of a pial vessel to treat an intracranial meningioma using Onyx.

## Introduction

Preoperative embolization of intracranial lesions has been widely accepted as a safe adjunct for minimizing operative morbidity. In most cases involving meningiomas, blood supply from branches of the middle meningeal artery is the primary target. Embolization of the intracranial supply has an inherently higher risk of ischemic injury. Furthermore, most available literature report the use of particles of particles (PVA) or Gelfoam pledgets as the embolic material [1–3]. In our case, we report treating a dysplastic aneurysmal branch within a meningioma that underwent embolization with Onyx.

## Case presentation

**Figure 1:**
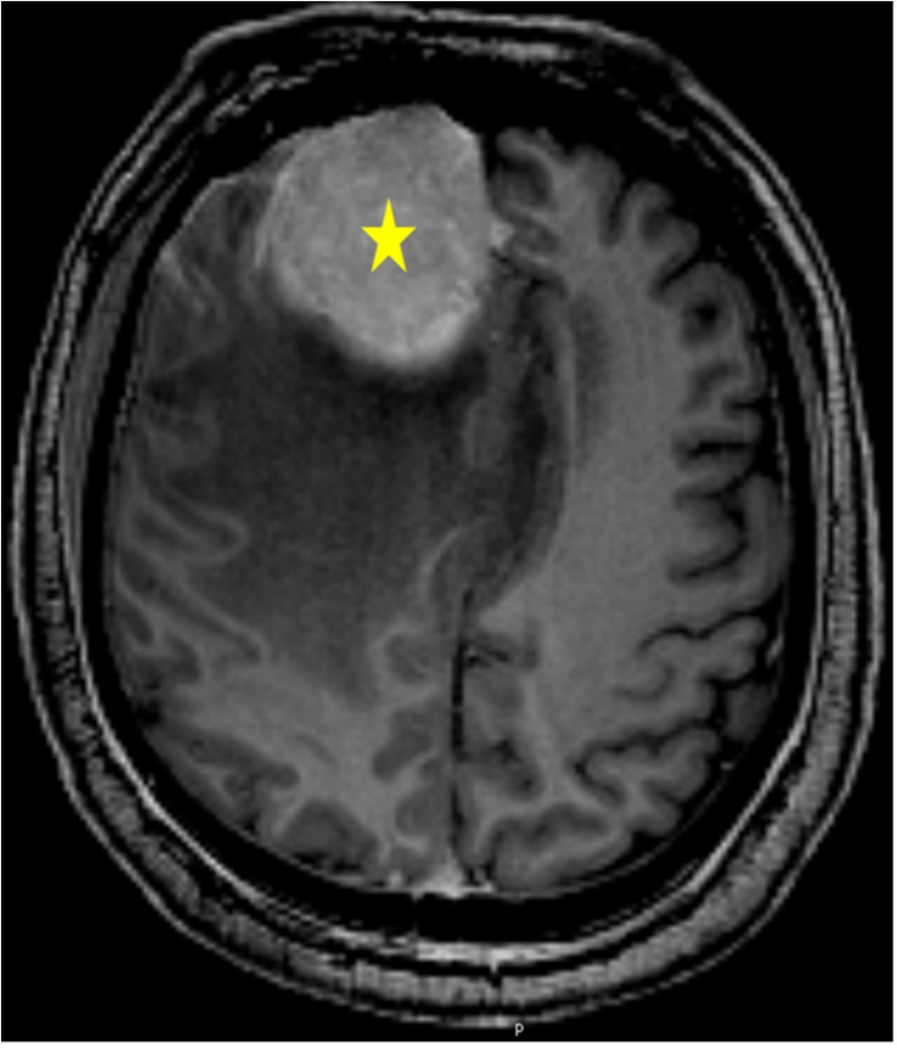
Axial Contrasted MRI MRI enhancing right parafalince extra-axial lesion (asterix) with surrounding edema and mass effect.

An elderly male with significant cardiac history presented with new-onset facial droop. A gadolinium-enhanced magnetic resonance image (MRI) was performed and is shown in Figure 1 below. It demonstrates an approximately 5 cm parafalcine meningioma as the underlying etiology.

**Figure 2:**
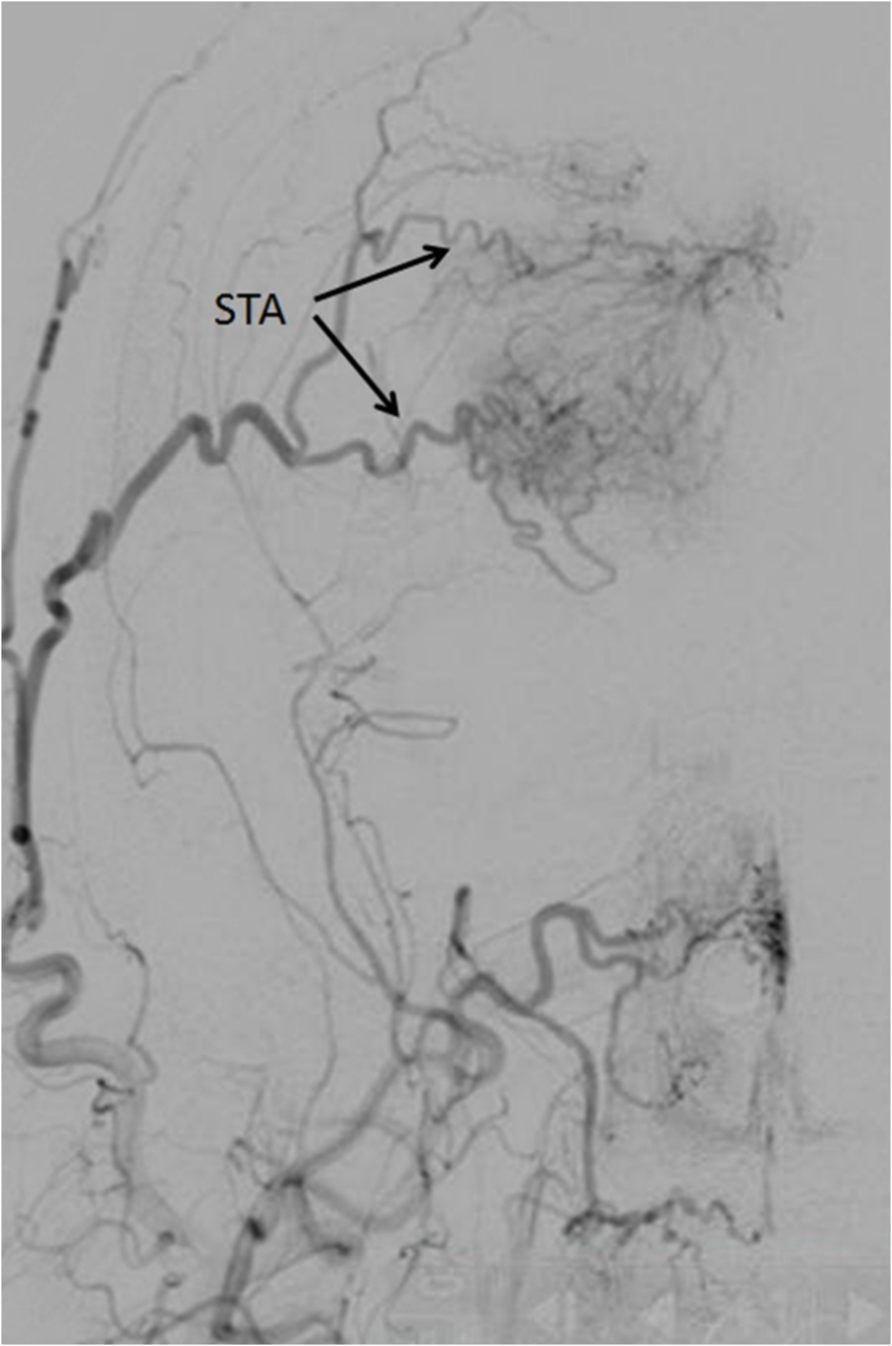
Right External Carotid Artery Injection Injection shows superficial temporal artery (STA) (arrows), arterialized feeders to the tumor.

Due to religious beliefs, the patient declined blood transfusion should it be needed. To minimize intraoperative blood loss, a preoperative angiogram was performed with the goal of embolizing the accessible blood supply to the tumor. Large arterialized feeders were noted from the right superficial temporal artery (STA) and left middle meningeal artery (MMA) as illustrated in Figure 2.

**Figure 3:**
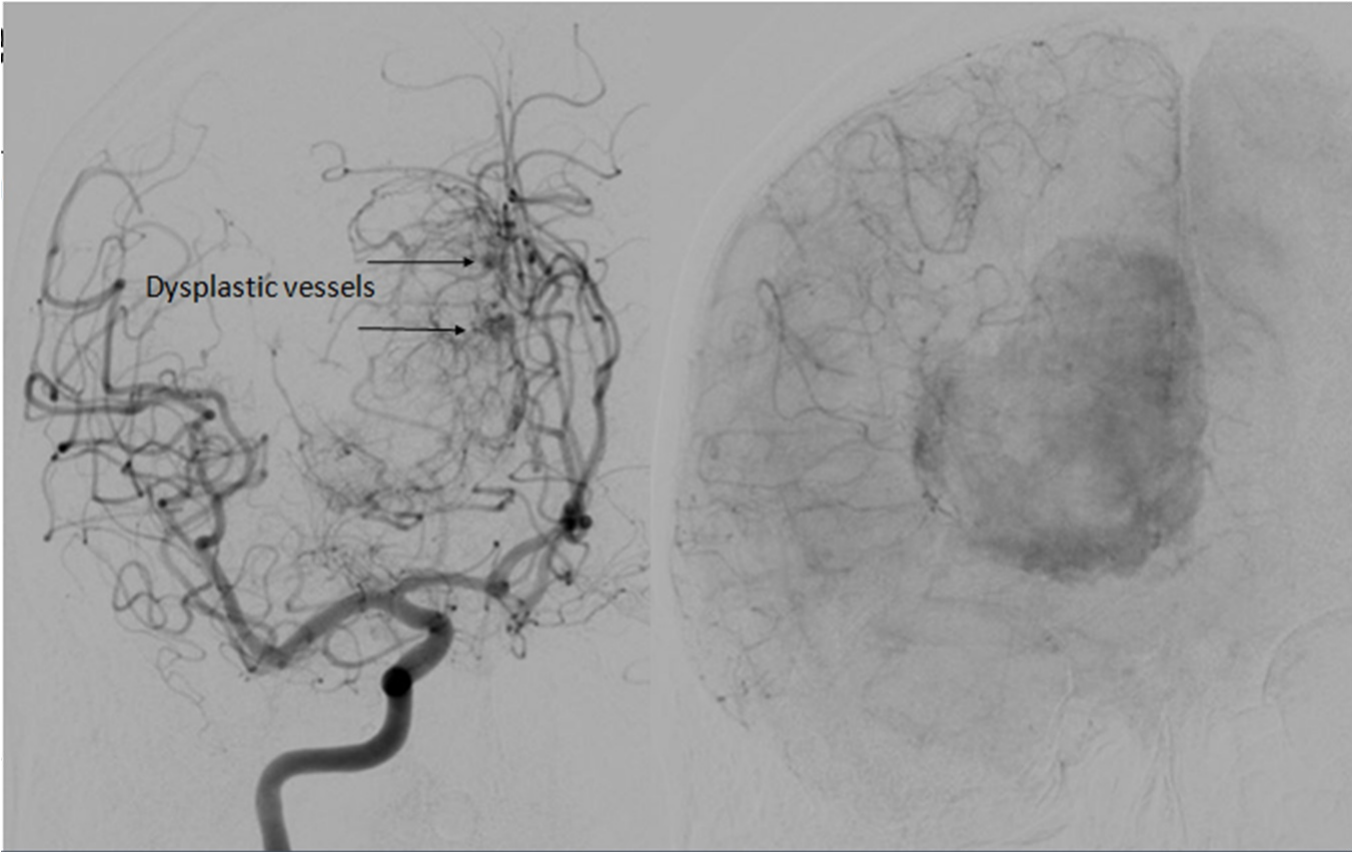
Right AP Internal Carotid Artery Injection Injection shows dysplastic ACA vessels also feeding the tumor.

Furthermore, Figure 3 shows several dysplastic-appearing vessels originating from the distal anterior cerebral artery (ACA) which provided vascular supply within the tumor itself.

**Figure 4:**
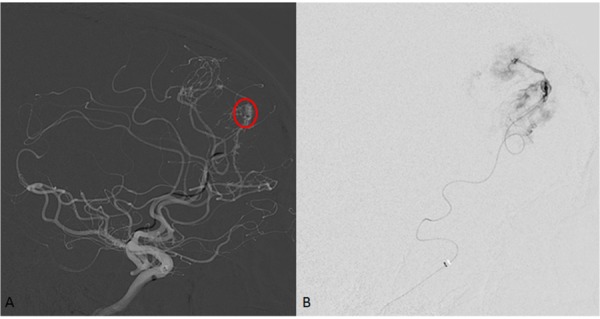
Selective ACA Branch 1 Injection Prior to Onyx Embolization A.  Lateral right roadmap view showing the microcatheter with the tip at the first dysplatic branch of the anterior cerebral artery. Tip is circled in red. B.  Digital subtraction view of the roadmap obtained in view A demonstrating the dysplatic branch prior to onyx embolization.

**Figure 5:**
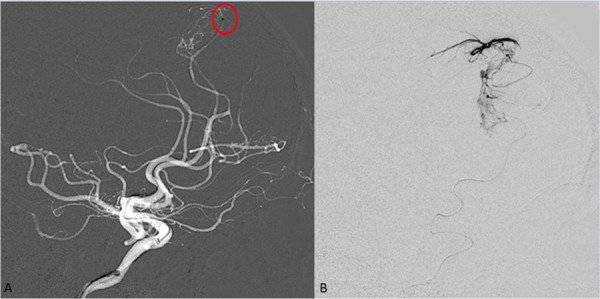
Selective ACA Branch 2 Injection Prior to Onyx Embolization A.  Lateral right roadmap view showing microcatheter with the tip at the second dysplastic branch of the anterior cerebral artery feeder to the tumor. Tip is circled in red. B.  Digital subtraction view of the roadmap obtained in view A demonstrating the second dysplastic branch off of the ACA prior to onyx embolization.

Upon further investigation, the one dysplastic vessel appeared to have an aneurysmal configuration that would have made the open microsurgical treatment more difficult. Given his circumstances, after treating the left MMA, the right ACA had two main dysplastic vessels that were superselected with a microcatheter using standard techniques. Under direct visualization, a total of 0.07 ml of Onyx-18 (ev3, Covidien) was injected into the two pathologic vessels (0.05 ml and .02 ml, individually) which are illustrated in Figure 4 and Figure 5, respectively.

**Figure 6:**
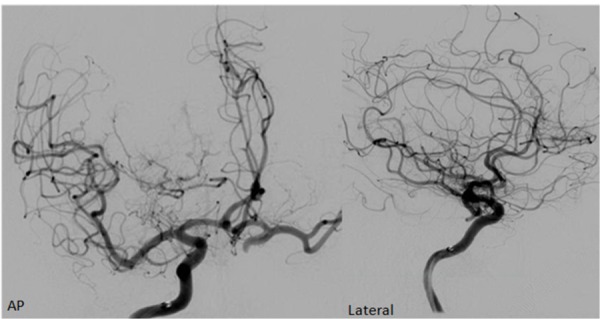
AP and Lateral Post-Onyx Angiography with Less Blood Flow to the Tumor

Post-treatment angiography as shown in Figure 6 confirmed significantly reduced blood supply to the lesion.

There were no complications associated with the procedure. The patient underwent standard microsurgical resection the following day without issues. Estimated blood loss was 150 ml, with special attention given to ligating the STA at the initiation of the procedure. The patient’s hospital course was uneventful with a resolution of his initial symptoms, and he was discharged on hospital day three. The patient agreed to participate and was explained the nature and objectives of this study, and informed consent was formally obtained. No reference to the patient's identity was made at any stage during data analysis or in the report.

## Discussion

Preoperative embolization of external carotid feeders to meningiomas to reduce operative morbidity is a widely accepted treatment option. Most embolization therapy commonly targets the supply provided by the middle meningeal artery, ascending pharyngeal artery, or occipital artery [1-3]. Furthermore, the most commonly employed embolization agents used are polyvinyl alcohol (PVA) particles, large-caliber microspheres, or coil occlusion. More recently, ethylene-vinyl alcohol (Onyx; Covidien, Irvine, CA) has been employed. These endovascular techniques provide an attractive adjunct therapy to treat vascular intracranial tumors by aiding in the extent of resection, altering tumor consistency, decreasing operative blood loss, and operative duration. This can be attained with minimal morbidity associated with the embolization procedure. In a recent single institutional experience, there was a 2.9% complication rate, without hemorrhagic transformation or cerebral edema due to the endovascular intervention [2]. This is similar to reported rates in the literature. In the single institution study, pial supply was not the target in the 224 patients treated due to a higher rate of ischemic complications. Overall, the goal of embolization is to make open surgery safer, which was the result in this case. There have been isolated reports regarding endovascular treatment of pial vasculature [4-7]. This is the first reported case of treating a dysplastic vessel providing pial supply with Onyx embolization. Given the robust vascular blush with shunting present, Onyx-18 was elected to achieve better intra-tumoral penetration to allow for decreased vascular supply. In conclusion, we report a rare combination of an intratumoral dysplastic vessel with aneurysmal features safely treated by Onyx embolization. Although the preoperative patient circumstance required a more aggressive than usual endovascular treatment, we report the potential validity of treating intra-tumoral pial vascular supply with Onyx. Further larger scale studies are warranted to investigate the safety of performing this treatment.

## Conclusions

A relatively common pathological entity, a parafalcine meningioma, revealed a rarely associated intratumoral dysplastic aneurysmal vessel from the intracranial vasculature. Given the patient’s religious preference, an aggressive endovascular intervention was merited. Onyx embolization via the anterior cerebral artery supply was safely performed with Onyx-18. Safety of intraoperative microsurgical resection with minimal blood loss was aided by uncomplicated pre-operative embolization.
